# Health related quality of life in adolescents with chronic fatigue syndrome: a cross-sectional study

**DOI:** 10.1186/s12955-015-0288-3

**Published:** 2015-07-03

**Authors:** Anette Winger, Gunnvald Kvarstein, Vegard Bruun Wyller, Mirjam Ekstedt, Dag Sulheim, Even Fagermoen, Milada Cvancarova Småstuen, Sølvi Helseth

**Affiliations:** Institute of Nursing, Faculty of Health Sciences, Oslo and Akershus University College of Applied Sciences, Postboks 4 St. Olavs plass, NO-0130 Oslo, Norway; Department of Clinical Medicine, UIT The Arctic University of Norway, Tromso, Norway; Institute of Clinical Medicine, Medical Faculty, University of Oslo, Oslo, Norway; Department of Pediatrics, Oslo University Hospital, Oslo, Norway; Department of Pediatrics, Akershus University Hospital, Lørenskog, Norway; Department of Pediatrics, Innlandet Hospital Trust, Lillehammer, Norway; KTH, Royal Institute of Technology, School for Technology and Health, Stockholm, Sweden; Center for Shared Decision Making and Collaborative Care, Oslo University Hospital, Oslo, Norway

**Keywords:** adolescents, chronic fatigue syndrome, CFS/ME, health related quality of life, public health nurse, and pediatrics

## Abstract

**Aim:**

To study health related quality of life (HRQOL) and depressive symptoms in adolescents with chronic fatigue syndrome (CFS) and to investigate in which domains their HRQOL and depressive symptoms differ from those of healthy adolescents.

**Background and objective:**

Several symptoms such as disabling fatigue, pain and depressive symptoms affect different life domains of adolescents with CFS. Compared to adolescents with other chronic diseases, young people with CFS are reported to be severely impaired, both physiologically and mentally. Despite this, few have investigated the HRQOL in this group.

**Method:**

This is a cross-sectional study on HRQOL including 120 adolescents with CFS and 39 healthy controls (HC), between 12 and 18 years. The Pediatric Quality of Life Inventory™, 4.0 (PedsQL) was used to assess HRQOL. The Mood and Feelings Questionnaire assessed depressive symptoms. Data were collected between March 2010 and October 2012 as part of the NorCAPITAL project (Norwegian Study of Chronic Fatigue Syndrome in Adolescents: Pathophysiology and Intervention Trial). Linear and logistic regression models were used in analysis, and all tests were two-sided.

**Results:**

Adolescents with CFS reported significantly lower overall HRQOL compared to HCs. When controlling for gender differences, CFS patients scored 44 points lower overall HRQOL on a scale from 0–100 compared to HCs. The domains with the largest differences were interference with physical health (B = −59, 95 % CI −54 to −65) and school functioning (B = −52, 95 % CI −45 to −58). Both depressive symptoms and being a patient were independently associated with lower levels of HRQOL

**Conclusion:**

The difference in HRQOL between CFS patients and healthy adolescents was even larger than we expected. The large sample of adolescents with CFS in our study confirms previous findings from smaller studies, and emphasizes that CFS is a seriously disabling condition that has a strong impact on their HRQOL. Even though depressive symptoms were found in the group of patients, they could not statistically explain the poor HRQOL.

## Background

Chronic fatigue syndrome (CFS) is a well-known, disabling and long-lasting condition with an estimated prevalence of 0.1 % to 1.0 % in children and adolescents (Nijhof et al. 2011, Crawley et al. 2011). The criteria from the Centers for Disease Control and Prevention (CDC), are most frequently used [1], and are also cited as the Fukuda-criteria. CFS is characterized by an unexplained disabling chronic or relapsing physical and mental fatigue of new onset lasting for more than six months, which is not relieved. It is combined with four or more of the eight specified accompanying symptoms: headache, muscle pain, joint pain, sore throat, tender lymph nodes, impaired memory or concentration, unrefreshing sleep, and malaise after exertion [1]. For children and adolescents, specific pediatric criteria have been introduced, requiring only three months of fatigue and only one additional symptom [2, 3]. Both pain and fatigue have great impact on physical activity [4], which again might act as a disease-maintaining factor [5]. Young people with CFS have reported more pronounced impairment, with reduced school attendance, compared with a corresponding sample of juvenile idiopathic arthritis [6]. This may be critical, as adolescents are in a vulnerable stage of life when social interaction with peers is highly important [7]. Depression is shown to be common in this group, but the causal relationship between CFS and depression is still unknown [8]. More important in this setting, anxiety and depression seem to be intensified by social isolation [9]. A previous qualitative study among healthy adolescents has shown that friends are one of the most important factors for HRQOL [10]. In the recent qualitative study from our group, the adolescents with CFS described a feeling of being different and forgotten when not being able to interact with friends or attend school [11]. CFS affects several life domains, and in adult CFS samples HRQOL has shown to be poor [12, 13]. However, few studies have thoroughly investigated the HRQOL perspective among adolescents with CFS. In a small study, including 25 children with CFS, Kennedy et al. [14] found that HRQOL was significantly more reduced compared with children and adolescents suffering from other chronic conditions.

Health-related quality of life (HRQOL) is a multidimensional construct consisting of different domains from physical, psychological, social and spiritual issues [15]. The functional capacity is affected in adolescents with CFS, but there is a lack of knowledge about physical impairment and how the impairments affect their HRQOL. When Kennedy et al. [14] assessed HRQOL among 25 children with CFS, they found physical health to be the most seriously affected domain. In other studies on this group, both physical and psychological functioning have been found to be particularly poor [16]. Our research group found that adolescents with CFS have a reduced level of activity measured as reduced number of steps daily [17]. This may have led to increased body mass index (BMI) [18] and poor physical quality of life [19].

The aims of this paper were to examine HRQOL and depressive symptoms in adolescents with CFS and to compare HRQOL and depressive symptoms with a group of healthy adolescents. We hypothesized that adolescents with CFS report lower HRQOL and have a higher degree of depressive symptoms compared to HCs.

## Material and methods

### Design

This is a cross-sectional study which describes HRQOL in adolescents with CFS and compares the HRQOL with a group of healthy adolescents. The study is part of the NorCAPITAL-project (The Norwegian Study of Chronic Fatigue Syndrome in Adolescents: Pathophysiology and Intervention Trial; Clinical Trials ID: NCT01040429) which explores possible mechanisms of CFS, the effect of low-dose clonidine treatment, and patients’ experiences in adolescents with CFS [17].

### Study population

#### CFS patients

One hundred and twenty adolescents with CFS and 39 healthy adolescents were recruited between March 2010 and October 2012. All pediatric departments in Norwegian hospitals (*n* = 20), as well as primary care pediatricians and general practitioners, were invited to refer adolescents with CFS (aged 12–18 years) to a central Norwegian department of pediatrics. The referring units were required to confirm that the patients did not have any medical or psychiatric disorder that might explain the fatigue. In agreement with clinical guidelines [2, 3] a “broad” case definition with three months of unexplained, disabling fatigue of new onset was required. We also required that the patient was not permanently bedridden and did not use pharmaceuticals (including hormone contraceptives) regularly. Those who fulfilled the pre-specified criteria for inclusion (Table 1), went through a thorough medical examination before they were invited to take part in the Nor CAPITAL study.

**Table 1:** Criteria for inclusion and exclusion

	CFS patients	Healthy control subjects
Inclusion criteria:	Persisting or constantly relapsing fatigue lasting 3 months or more.	Age ≥ 12 years and < 18 years
	Functional disability resulting from fatigue to a degree that prevent normal school attendance	
	Age ≥ 12 and < 18 years	
Exclusion criteria:	Another current process or chronic disease or demanding life event that might explain the fatigue	Another chronic disease
		Permanent use of pharmaceuticals (including hormones)
	Permanent use of pharmaceuticals (including hormones) possibly interfering with the measurements	
	Permanently bed-ridden	
	Positive pregnancy test	
	Pheocromocytoma	
	Evidence of reduced cerebral and/or peripheral circulation due to vessel disease	
	Polyneuropathy	
	Renal insufficiency	
	Known hypersensitivity towards clonidine or inert substances (lactose, saccarose) in capsula	
	Abnormal ECG (apart from ectopic beats)	
	Supine heart rate < 50 beats/min	
	Supine systolic blood pressure < 85 mmHg	
	Upright systolic blood pressure fall > 30 mmHg	

The criteria are designed for the randomized control trial in the NorCAPITAL-project (The Norwegian Study of Chronic Fatigue Syndrome in Adolescents: Pathophysiology and Intervention Trial; Clinical Trials ID: NCT01040429), which explores possible mechanisms of CFS, the effect of low-dose clonidine treatment, and patients’ experiences as adolescents with CFS (9)

#### A control group of healthy adolescents

To recruit a control group of healthy adolescents, information about the study was sent to local schools. Those who replied, were given extended information. No regular use of pharmaceuticals (including hormones) was required to be included. A group of 39 healthy and normally active adolescents and comparable on gender and age, was enrolled. The HCs, however, were not actively matched.

### Measures

#### Body Mass Index

Research in children and adolescents shows that overweight and obesity have impact on their HRQOL [20]. According to The Norwegian Directorate of Health [21], BMI is calculated as weight in kilograms divided by height in squared meters and is adjusted for age and gender. We decided to transform BMI into a categorical variable with three categories; BMI < 25 (normal weight), 25 ≤ BMI <30, and BMI ≥ 30 (Table 1). Weight and height were measured by the researchers.

#### PedsQL

To measure HRQOL we used the Pediatric Quality of Life Inventory™, 4.0 (PedsQL), which is a 23-item generic questionnaire developed to measure HRQOL in healthy children and adolescents, as well as in pediatric populations with acute and chronic health conditions [22]. The questions cover all the health domains stated by WHO including school functioning [23] and are suitable for children and adolescents aged between 5 and 18 years. Corresponding questionnaires for proxies exist (there are several versions dependent on age). The PedsQL provides a total sum score and four subscale scores in the following domains; (1) physical functioning (8 items), (2) emotional functioning (5 items), (3) social functioning (5 items), and (4) school functioning (5 items). Three of the subscales (emotional, social, and school functioning) are combined to provide a specific scale for psychosocial health. Participants are asked to rate the severity of each item during the previous month by a Likert Scale from 0 (never a problem) to 5 (a lot of a problem). Items are reverse-scored and linearly transformed on a scale ranging from 0–100 (0 = 100, 1 = 75, 2 = 50, 3 = 25, 4 = 0), where higher scores indicate better HRQOL. Each subscale score is computed as the sum of points from each item divided by the number of items answered [23]. The instrument has been translated into several languages, among these Norwegian, and validated [24]. Scale internal consistency was measured with Cronbach’s alpha, varying between 0.77 and 0.88, which is considered good [24].

#### The mood and feelings questionnaire

Levels of depressive symptoms were identified using The Mood and Feelings Questionnaire (MFQ) which is validated for children and adolescents [25]. MFQ consists of 33 items with three alternative answers (not true = 0, sometimes = 1, true = 2). Total score ranges from 0 to 66. A score ≥ 20 implies presence of depressive symptoms to a degree that suggests mood disorder [25], and was therefore chosen as the cutoff. MFQ has been used in several Norwegian studies [26–28], showing good internal consistency [26].

Also the PedsQL includes questions concerning depression-like symptoms. These questions, however, are phrased differently and cover slightly different areas compared to questions in MFQ. Moreover, the questions covering depressive symptoms, are to be scored differently, and represent only a part of the overall score. We thus found it meaningful to model associations between HRQOL and depressive symptoms.

### Ethical considerations

The study was approved by the Norwegian Social Science Data Service and by the Norwegian Regional Committee for Medical and Health Research Ethics. Participation in the project required informed consent by the adolescent and by his or her parents/next-of-kin after written and oral information about the study.

### Statistical analyses

Continuous data (age and disease duration) are presented as means with *SD* while categorical data (gender, parents’ education and BMI) are presented as numbers and percentages. Differences between CFS patients and HCs were assessed with Chi-square test analyses for the independent categorical variables gender and BMI.

To further investigate the differences between CFS patients and HCs, we fitted a linear regression model with overall HRQOL score and the sub-scores for each domain as dependent variables. We moreover adjusted for gender, depressive symptoms and categories of BMI. The factor BMI, however, did not reach statistical significance and was excluded from further analysis. The risk of having depressive symptoms (a dichotomized dependent variable) was modeled using logistic regression and adjusted for gender and BMI.

Both regression models fitted the data well (goodness of model fit), and all residuals were approximately normally distributed (linear regression). The results are presented as estimates of beta (B) with 95 % confidence intervals (CI). The results of logistic regression are presented as odds ratios (*OR*) with 95 % CI. All tests were two-sided, and *P*-values <0.05 were considered statistically significant. All analyses were performed using IBM SPSS statistics version 21.

There were almost no missing data in any of the data sets. Values were imputed for individuals when less than 10 % of the values were missing by using the mean score for each variable. Individuals who had more than 10 % missing values in total, were excluded.

As a result of difficulties in recruitment of healthy controls, our sample of HCs was smaller than the group of adolescents with CFS. We therefore compared the average levels of the HCs with corresponding levels in the healthy population in the Norwegian validation study of the PedsQL [24], and the control group of HCs in the study by Diseth and coworkers [29] including children/adolescents subjected to renal transplantation (TX) and children in remission after acute lymphoblastic leukemia (ALL). Confidence intervals were not available in these studies, but knowing the mean and *SD*, we could compute the CI making it possible to compare the data.

## Results

In total, we included 120 adolescents with CFS and 39 healthy adolescents as controls. Participants were comparable regarding age (mean 15.4 for CFS patients and mean 15.2 for HCs), and gender (72 % girls in both the CFS and HC group).

The comparison of our relatively small group of HCs with other studies using PedsQL to measure HRQOL in children, suggest that our sample of HCs is a representative control group (Table 2).

**Table 2:** Comparing PedsQL results from the NorCAPITAL study with two other studies

	NorCAPITAL study				Reinfjell et al. 2006 [24]		Diseth et al. 2011 [29]		Diseth et al. 2011 [29]		Diseth et al. 2011 [29]	
	CFS	95 % CI	HCs	95 % CI	HCs	95% CI	HCs	95 % CI	TX	95 % CI	ALL	95 % CI
	Mean (SD)		Mean (SD)		Mean (SD)		Mean (SD)		Mean (SD)		Mean (SD)	
Variable	N = 120		N = 39		N = 425		N = 42		N = 38		N = 40	
Age	12-18		12-18		13-15		3-19		3-19		3-19	
Overall QoL	49 (13)	46-51	93 (8)	89-96	85 (11)	84-86	89 (8)	87-91	69 (18)	63-75	82 (13)	78-86
Physical health	37 (17)	33-40	96 (8)	92-99	91 (10)	90-92	92 (6)	90-94	75 (17)	69-81	86 (12)	82-90
Emotional functioning	60 (18)	56-63	88 (14)	83-94	77 (17)	75-79	83 (13)	79-87	69 (16)	61-74	75 (19)	69-81
Social functioning	70 (15)	68-73	98 (4)	97-99	88 (13)	87-89	93 (8)	90-96	74 (22)	67-81	86 (14)	82-90
School functioning	36 (19)	33-39	88 (14)	81-93	78 (15)	77-79	86 (13)	82-90	63 (18)	57-69	77 (16)	72-82
Psychosocial functioning	57 (15)	54-60	91 (10)	88-96	82 (13)	81-83	87 (9)	84-90	67 (18)	61-73	79 (14)	75-83

HRQOL is assessed by PedsQL

Almost all the patients had suffered from CFS for a long period of time (mean 21.4 months) (Table 3), and only two patients had a short disease duration of between three and six months.

**Table 3:** Demographic data and adherence to the CDC-criteria for CFS

	CFS patients	Controls
	N = 120	N = 39
Variable	n (%)	n (%)
Male	34 (28)	11 (28.2)
Female	86 (72)	28 (71.8)
CDC criteria	88 (74)	NA
Lives together with both parents	85 (73)	26 (70)
BMI (adjusted for age and gender)		
Normal weight (BMI <25)	100 (83.3)	37 (94.9)
Moderate overweight (25 ≤ BMI < 30)	14 (11.7)	2 (5.1)
Severe overweight (BMI ≥30)	6 (5.0)	0 (0)
MFQ (depressive symptoms)^a^	47 (39)	3 (8)
Parents highest education		
Primary school	5 (4.3)	0 (0)
Secondary school	30 (26)	8 (23)
Lower university	34 (29)	9 (23)
Higher university	48 (41)	19 (54)
	Mean (SD)	Mean (SD)
Age	15.4 (1.6)	15.2 (1.6)
Disease duration (months)	21.4 (15.2)	NA
School absence %^b^	65 (30)	2.1 (6.8)

^a^MFQ (Mood and feelings Questionnaire) score >20 suggests depressive symptoms and was the cutoff chosen. NA = not applicable. ^b^School absence was calculated as the ratio between mean days absent from school last month and days supposed to be at school last month. These proportions are presented as mean values with SD

As there was no difference between the CFS group and HCs concerning demographics such as parents’ education (Table 3) and ethnicity (data presented elsewhere [17]), we did not need to adjust for these variables when assessing group differences. The CFS group had a slightly higher mean BMI, but the group difference did not reach statistical significance.

**Fig. 1:**
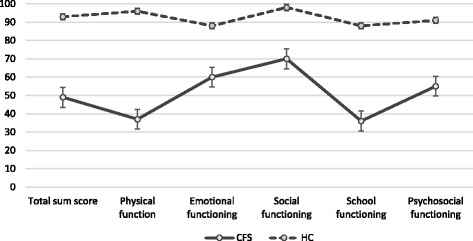
Health Related Quality of Life among Adolescents with CFS and Healthy Controls. *(Detailed Legend Fig.* 1 *):* HRQOL was assessed by the Pediatric Quality of Life Inventory™, 4.0 (PedsQL). Total score and subscales scores are presented by mean values and SD. CFS: N: =114, HCs: N = 34

When adjusted for gender, CFS patients had significantly lower overall HRQOL than did the HCs (44 HRQOL points, B = −44, 95 % CI (−39.6 to −48.6)) (Table 4), and for each subscale the group differences were even more pronounced; on a scale from 0–100 it was 60 points lower for physical functioning, 52 points lower for school functioning, 28 points lower for emotional functioning, and 27 points lower for social functioning. Concerning psychosocial HRQOL, the CFS patients had on average 33 points lower scores than HCs, independent of gender and BMI (Fig. 1 and Table 5).

**Table 4:** Comparison of health related quality of life between adolescents with CFS and HCs

Variable	Items	Estimate of B	95 % CI	*p*-value
Overall HRQOL	23	−44	(−49; −40)	<0.001
Physical health	8	−60	(−54; −66)	<0.001
Emotional functioning	5	−28	(−22; −35)	<0.001
Social functioning	5	−27	(−22; −33)	<0.001
School functioning	5	−52	(−45; −58)	<0.001
Psychosocial functioning	15	−36	(−31; −41)	<0.001

HRQOL was assessed by the Pediatric Quality of Life Inventory™, 4.0 (PedsQL). The listed estimates express the difference between adolescents with CFS and healthy controls. Multiple linear regression for total score and subscale scores are adjusted for gender differences between the groups. CFS: *N*: =114, HHCs: *N* = 36

**Table 5:** Health related quality of life among adolescents with CFS and HCs

	Scores, Mean (SD)	Scores, Mean (SD)
	Adolescents with CFS	Healthy Controls
	(n = 114)	(n = 36)
Overall HRQOL^a^	48.8 (13.0)	92.9 (8.2)
Physical health	36.5 (17.2)	96.0 (8.1)
Emotional functioning	59.8 (18.2)	88.1 (14.0)
Social functioning	70.0 (15.1)	97.8 (3.9)
School functioning	36.0 (18.8)	87.6 (13.9)
Psychosocial functioning^b^	55.4 (13.1)	91.2 (8.9)

HRQOL was assessed by the Pediatric Quality of Life Inventory™, 4.0 (PedsQL)

^a^Sum score of all questions. ^b^Combined score of emotional, social and school functioning. Total score and subscales scores are presented as mean values with SD

Figure 1 depicts point-estimates for HRQOL for CFS patients and controls with standard errors of the mean values for sum-score and sub-scores for each domain. The estimates show a high precision due to the high number of CFS patients and the homogeneity of the control group. The specific values are presented in Table 5.

Regarding gender differences, girls (both adolescents with CFS and HCs) demonstrated a 5 points lower overall HRQOL level (B = −4.7, 95 % CI (−8.9 to −0.5)) compared to boys. This was also the case for the subscales physical health (B = −5.9, 95 % CI-11.3 to −0.5) and emotional functioning (B = −7.3, 95 % CI −13.4 to −1.3).

Logistic regression analysis indicated an eight times higher risk of depressive symptoms when having CFS compared to HCs (*OR* = 7.9, 95 % CI 2.3-27.4). When modeling quality of life using linear regression, higher levels of depressive symptoms were inversely associated with higher levels of HRQOL both in CFS patients and in HCs. Further, we modelled HRQOL with multiple linear regression. When depressive symptoms was entered as a covariate, we only uncovered an additive effect of depressive symptoms and no interaction between having a higher level of depressive symptoms and being a CFS patient. Thus having a higher level of depressive symptoms was associated with the same change of effect on HRQOL in CFS patients and HCs, and the strength and size of the association between being a CFS patients and HRQOL were not changed when adjusted for depressive symptoms. We therefore conclude that both depressive symptoms and being a patient were independently associated with lower levels of HRQOL.

## Discussion

Our findings show that adolescents with CFS have a significantly lower quality of life compared with healthy controls, demonstrated by lower overall HRQOL score and sub-score levels for specific HRQOL domains. Depressive symptoms were found in both adolescents with CFS and HCs, but the score levels were higher among the adolescents with CFS. The low HRQOL level in the CFS group was not explained by depressive symptoms but by having CFS.

### Health related quality of life and depressive symptoms

This is the first large study investigating HRQOL in adolescents with CFS. Both overall HRQOL and the subscale-scores for different life domains were lower compared to HCs, in line with the results from a smaller, previous study which included 25 adolescents with CFS [14]. The group differences in our study were not explained by gender, BMI or socio-demographic differences.

Although depressive symptoms is found to be the main predictor for reduction in daily functioning in adults with CFS [30], the cause effect relation between CFS and depression is still debated [8]. Skapinskis et al., studying an adult population, proposed that depression might lead to CFS [31], while Bould and coworkers, on the contrary, suggested that the experience of having CFS could explain depressive traits in adolescents with CFS [32]. The CFS patients in our sample showed an eight times higher risk of having a mood disorder, but a higher level of depressive symptoms had statistically the same effect on HRQOL in both groups. Our model showed that having depressive symptoms and having CFS independently explained the low HRQOL scores. Thus, being a CFS patient was an independent factor explaining the lower HRQOL, and it was not confounded by depressive symptoms.

We chose a low cut-off indicating mood disorders and not a clinical relevant cut-off screening for major depression. This might be the reason for the relatively high proportion of HCs (8 %) with depressive symptoms.

### Emotional functioning

On emotional functioning, our CFS population scored 60 out of maximum 100 points; 30 points lower than the group of healthy adolescents. Even though we used a different questionnaire, the results are almost equal to Kennedy et al. who found 54 points out of 100 on emotional function. Alexithymia, which means difficulties in experiencing, expressing, and describing emotional responses, has been suggested as a factor in the development of medically unexplained diseases such as CFS. In a study by van de Putte et al. [33], however, alexithymia did not correlate as a unique factor to CFS and did not appear as a prognostic factor for recovery from CFS. A study by Garralda & Rangel [6] has shown that children with CFS use more emotional regulation or resignation and fewer problem-solving strategies to deal with disabilities compared with other children. Experiencing difficult thoughts and sad feelings (depressive symptoms) might not be surprising, considering the consequences of the disease, such as reduced school attendance and time with peers. Thus, it should be of importance to support the adolescents in handling sad and difficult feelings.

### Physical functioning

Despite the sparse number of studies on HRQOL in adolescents, those which are available address domains like physical impairment and school attendance [34, 4, 35]. It should be borne in mind that physical health in PedsQL includes both physical activity, the feeling of low energy, and pain as an unpleasant bodily symptom [23], as they are all interconnected. Low mood and fatigue are associated with worse physical function and are the strongest predictors for school absence [4].

The fact that physical health and school functioning were most affected in our sample, might not be surprising considering the nature of CFS. Garralda and Rangel (2004) have demonstrated that children (10–18 years) with CFS are even more impaired compared with children with juvenile idiopathic arthritis (9–18), especially when it comes to schoolwork, expectations and attendance. This was also the case when compared with children with type 1 diabetes and asthma [14]. We further compared our findings (Table 2) with a recent Norwegian study on HRQOL including children and adolescents subjected to renal transplantation (TX) (with a median age since transplantation at 4.9 years), and children in remission after acute lymphoblastic leukemia (ALL) [29]. Our study suggests that adolescents with CFS were even more interfered by their illness than adolescents with renal TX and ALL. We have previously reported that our CFS patients performed a significantly lower number of steps per day [17], suffered from more severe pain, and had a lower pressure pain threshold than HCs [36]. The low activity among CFS patients might stem from the feeling of extreme fatigue and/or activity-related pain. When experiencing malaise and pain after even normal activity, patients may easily develop a fair avoidance behavior [35] as demonstrated among adults with CFS [37]. Gray and Rutter [38] identified two important factors for predicting physical functioning in adolescents with CFS: adolescents who adapted to the illness (using Leventhal’s self-regulation model measuring) reported a better quality of life, and those who managed to maintain some degree of activity reported better physical outcomes. A stronger focus on symptoms was by contrast associated with poorer HRQOL. The authors suggested that adapting to the illness might act as a mediator, leading to better coping strategies.

### School functioning as a factor for social interaction

In our study, school functioning was the most affected HRQOL domain, with a score level (Fig. 1, Table 3) substantially lower compared with previous studies on CFS patients [14, 39, 6]. In a qualitative study, recently published, we explored adolescents’ own experience of living with CFS, and found that lack of participation at school and in teen venues made them feel like outsiders [11]. This is a serious finding, considering that a good relation to friends is one of the most important factors for HRQOL in healthy adolescents [10]. Other studies on adolescents with CFS have similarly emphasized the need for friends as an important factor for well-being [9, 40]. The adolescents with CFS experienced loss of a normal life, and the changes in friendships as difficult, leading to loneliness and isolation [9].

School is surely an institution for learning and intellectual activities, but is also an arena for socializing. Previously, we have reported that some of the adolescents managed to shift focus from friends and school towards other arenas like their family, and appreciated their support [11]. Thus, relational factors within the family seem to be even more important for children with CFS than those with other chronic conditions [41]. In this way, however, adolescents with CFS might become even more dependent on care-givers [35] which may hinder growing towards independence. This dilemma underpins the importance of involving the family in treatment. The dramatic changes following low school functioning may have serious educational and social implications [4]. A study on school drop-outs in adolescents with chronic somatic diseases found an increased risk of sickness and disabilities later in young adulthood [42]. To minimize school drop-out and the risk of long term consequences, the authors pointed to the importance of early intervention.

### HRQOL and gender differences

Looking at gender difference, the overall scores of HRQOL were lower among girls compared to boys, both in the CFS and the HC group. Gender differences have also been demonstrated in previous HRQOL studies [24] and are not unique in adolescents with CFS.

### Strengths and limitations

The Nor CAPITAL study had one clear selection bias, as the study included only patients who were able to attend our research clinic; and the results can therefore not be extrapolated to the most seriously affected CFS adolescents. Secondly, the number of HCs in our study was only one third compared to the group of CFS patients. We therefore additionally compared the average levels of the HCs with corresponding levels from the healthy population in the Norwegian validation study of the PedsQL [24] and the control group of HCs in a study by Diseth and coworkers [29] (Table 2). The comparison with the other studies suggest that our sample of HCs is a representative control group.

Validating MFQ, Daviss et al. [25] suggested two different cutoffs which differentiate between depressive symptoms (cutoff ≥20), and major depression (cutoff ≥29). The relatively high proportion of patients with depressive symptoms in our study might be explained by the low cutoff value. Although depressive symptoms were only found to have an additive effect on HRQOL, this does not change the fact that adolescents with CFS report seriously poor HRQOL.

### What this study adds

The large study population contributes to underpin an area almost not studied and documented in this population. The study provides new knowledge about how bad the life of these adolescents are, and their HRQOL was even poorer than we expected. The poor HRQOL was explained by the illness and not by depressive symptoms. The relatively large study population with few missing data makes it possible to generalize the findings.

## Conclusion

The large sample of adolescents with CFS in our study confirms previous findings from smaller studies and emphasizes that CFS is a seriously disabling condition that has strong impact on their HRQOL. Compared to healthy adolescents, HRQOL in adolescents with CFS was even poorer than we expected, and physical function and school function showed the lowest sub-scores. Even though depressive symptoms were found in the group of patients, they could not statistically explain the poor HRQOL.
